# Study on the Quality Control for Periodogram in the Determination of Water Level Using the GNSS-IR Technique

**DOI:** 10.3390/s19204524

**Published:** 2019-10-17

**Authors:** Minfeng Song, Xiufeng He, Xiaolei Wang, Ye Zhou, Xueyong Xu

**Affiliations:** 1 School of Earth Science and Engineering, Hohai University, Nanjing 211100, China; smf@hhu.edu.cn (M.S.); gnsswxl@hhu.edu.cn (X.W.); 2 HORINCO North Information Control Research Academy Group CO.,Ltd, Nanjing 211100, China; Leaf430ship@126.com (Y.Z.); xxyyeah@163.com (X.X.)

**Keywords:** GNSS-IR, quality control, water level measurement, SNR, GQCS

## Abstract

A GNSS station, located on the shore of sea and inland waters, and equipped with standard geodetic receivers and antennas, can be used to measure water levels using a technique called GNSS Interferometric Reflectometry (GNSS-IR). The classical GNSS-IR method is based on SNR data and LSP spectrum analysis method. In order to promote the application of GNSS-IR, the accuracy of the results needs to be further improved, and quality control needs to be achieved better. Classical quality control methods include denoising filtering based on data source SNR; post-processing filtering based on results; morphological analysis based on parameters, such as the ratio of the maximum peak value to the background noise mean, the ratio of the maximum peak to the sub-peak, and the amplitude of the maximum peak. All three methods have the problem of correct frequency extraction under multiple approximate peak conditions. This paper focuses on the performance analysis of three methods of quality control for two situations with real examples, summarizes the advantages and disadvantages of each method, and discusses the measures in applications. Considering the limitations in the threshold setting for the third method, a new quality control method combining multiple parameters and external constraints is proposed. This method is more flexible, especially in dealing with a periodogram with multiple similar peaks, breaking through the premise that the frequency corresponding to the maximum peak is the correct frequency, and validated in two different environments. The experimental results show that the proposed method can improve the accuracy of the measured water level while ensuring the amount of the results. It eliminates the gross errors effectively and uses the data efficiently.

## 1. Introduction

Recent years have seen the development of a technology, called GNSS remote sensing, for monitoring ground surface environments, which uses GNSS reflected signals. It combines GNSS technology with remote sensing and becomes a new research area. There are two ways to combine direct and reflected signals of a GNSS satellite. One is based on signal-to-noise ratio (SNR), namely GNSS Interferometric Reflectometry (GNSS-IR) [1,2,3,4] and the other is based on Doppler Delay Map (DDM), namely GNSS-Reflectometry (GNSS-R) [5,6]. The former uses a geodetic GNSS receiver and antenna to obtain the interference signals by direct and reflected signals. The latter, however, requires a special antenna and receiver to receive two signals independently, and its principle is more complicated. This paper discusses the GNSS remote sensing technology based on the SNR to sense the changes of water level.

Many scholars have used the classical GNSS-IR technology to measure sea level changes and obtained effective results [7,8,9]. Several approaches have been proposed in the data processing, such as elevation angle correction [10], dynamic correction of reflecting surface [11]. Larson and Lofgren used ordinary geodetic receivers to monitor sea levels for the first time [12]. Experiments were carried out at the Onsala Space Observatory (OSO) in Sweden and the Friday Harbor GPS site in the United States. Compared with a special dual-antenna GPS tide gauge and took the measured data of tide gauge station near GNSS sites as the truth value, the RMS of the two methods were 5 cm and 10 cm, respectively. The correlation coefficient reached 0.97, but when the sea surface is rough due to strong wind, the dual-antenna GPS tide station will lose the useful data and dynamic monitoring of sea level becomes impossible. In this respect, the general geodetic receiver is of advantage. Larson [11] analyzed the one-year data of GPS stations near Kachemak Bay in Alaska in 2013, and proposed a dynamic correction model that can effectively correct errors due to the dynamic changes in the reflecting surface. The derived sea levels are compared with the measured data at a traditional tide station, 30 km away, and the RMS of daily mean sea level is 2.3 cm. Lofgren [2] analyzed the data at five GPS stations using the SNR data with the satellite elevation angles below 5°. Higher accuracy of the derived sea level with RMS from 6.2 cm to 43 cm, and correlation coefficient from 0.89 to 0.99 are obtained. Santamaría-Gómez [13] used the SNR data from both the L1 and L2 bands at eight GPS tide stations to gauge the sea level changes. The results are in good agreement with the tide station records and the accuracy is better than 3 cm. It is also found that when the signals from low elevation angles (below 12°) are used, systematic errors may occur, which can even reach to 15 cm. Larson [14] analyzed the 10-year GPS data at a station in Friday Harbor with the dynamic correction of the reflecting surface and the correction of the elevation angle refraction. The accuracy of a single measurement is 12 cm, and the accuracy of the daily average water level is 2 cm.

Quality control plays an important role in data processing. Whether it is denoising raw data, parameter control during data processing, or filtering denoising after data processing, it can further improve the accuracy and effectiveness of the results. Larson et al. [11] analyzed the data at the PBAY station to detect sea level, and proposed that the peak-to-noise ratio of periodogram should greater than 3, and “non-physical” reflector height peaks need to be discarded, and a normalized spectrogram peak of 1 dB-Hz. Santamaría-Gómez [13] proposed a quality control based on a priori estimates and using Kalman filtering algorithm in data processing. In the case of high sampling rate of GNSS receiver, this method can suppress the influence of SNR noise. Nievinski [15,16] proposed the use of degree of freedom, goodness of fit, reflector height uncertainty, peak elevation angle for quality control when using GNSS-IR technology for snow depth detection. The parameters are used to analyze the availability of SNR sequences from different aspects and subsequently to obtain good control effects.

Santamaría-Gómez [17] developed a new approach, using the extended Kalman filter/smoother algorithm to analyze the data of the PBAY station, and tilting the antenna to detect stronger interference effects, obtaining an accuracy of RMS of 3 cm, and found The L2(P) SNR sequence can exhibit a misleading double peak phenomenon, which affects the accurate extraction of frequencies. Based on the wavelet decomposition method, Wang [18,19] proposed a multi-layer decomposition combination method to remove the SNR noise, so that the combined wavelet base energy is closer to the idealized oscillation waveform, thus obtaining a cleaner period diagram. Wang [18,20] developed a state transition equation set and a robust regression solution strategy to formulate a 10-min sea level time series for sea level monitoring in complex environments, which improved the accuracy by nearly 40%–75%.

Strandberg [21,22] proposed a multi-parameter least squares fitting method to fit the data of signal-to-noise ratio after removing the trend, which can suppress some noises and get better results. But when the sampling rate of the receiver is low and the distance between the antenna and reflecting surface exceeds a certain limit, the detrended SNR will be more complicated. In this case, no periodic oscillations exist, and the fitted attenuation oscillation curve is quite different from the actual one, distorting the correct information and making extraction of the correct frequency information impossible. Therefore, the results obtained based on the fitted curve may also be poor.

However, few scholars have studied the water level measurements for inland water storage facilities based on the GNSS-IR. The deployment of inland freshwater resources and their rational use need the effective measurement of water levels. Compared with coastal GNSS sites, inland waters have special advantages for the accurate measurement of water level changes with GNSS-IR, because they have less water surface fluctuations, and the reflection of GNSS signals is closer to specular reflection, resulting in stronger reflection and more prominent interference effect. Xi [23] recorded the water level measurements of a reservoir by GNSS-IR technology for one year. Compared with the manual measurements, the correlation coefficient was above 0.93, which proved to be an effective supplement to the reservoir water level station. Moreover, the data was used in the deformation analysis, which broadens the application horizon of GNSS-IR technology in dam monitoring. However, due to the obstacles around the site, a large background noise can exist in the SNR time series. The method adopted is to strictly control the range of elevation angle and azimuth according to the relative position of the station and the water area. In addition, if the dominant peaks to rest of peaks in the periodogram does not exceed a threshold, the result will also be discarded. Since the development of GNSS reflected signal based on SNR data, the whole process and the accuracy of water level measurement has been gradually improved. However, many factors still limit the accuracy and temporal resolution in the water level determination, especially for sea. The environment of a GNSS site on a coast is more complicated, and there exist more than one reflecting objects, resulting in strong noise, which is not favorable for the data processing. The least square fitting method proposed in [21] is not suitable for the water level determination in a poor environment, but it is more reliable to analyze the original detrended SNR data. In this case, the quality control of the periodogram is crucial, because of multiple similar peaks and their small amplitudes in the periodogram. The quality control using traditional amplitude and signal-to-noise ratios does not effectively address all situations and will reduce data utilization and temporal resolution of the results. Although the inland waters have a better environment, a single quality control method due to its limitations and threshold setting which cannot respond to the changes in the environment, cannot be used for extracting and analyzing the effective frequencies. Therefore, it is necessary to develop an efficient and flexible quality control method for periodogram analysis. In this paper we first analyze the measurements of sea level at BRST station and reservoir water level at a dam monitoring station as examples, and then propose a flexible periodogram quality control method.

## 2. GNSS-IR Water Level Measurement Principle

**Figure 1:**
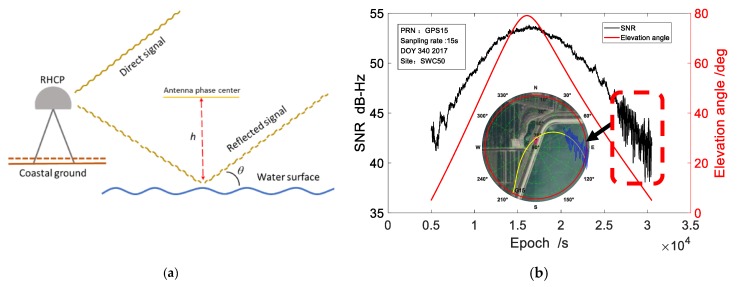
(**a**) Schematic diagram of GNSS-IR water level monitoring, (**b**) The relation between the signal-to-noise ratio and azimuth and elevation angle in the direction of water area.

Under suitable environments water levels can be measured by a geodetic GNSS receiver. For a GNSS stations on the shore of a water area, the signal-to-noise ratio (SNR) data of satellite signals, which are from a low elevation angle and towards the water direction, can be analyzed to obtain the vertical distance from the antenna phase center to the reflecting water surface, as shown in Figure 1a.

When a satellite is at a low elevation angle field and in a particular direction, due to the gain of the antenna and the strong reflection effect of the water surface, the antenna will receive an interference signal that is synthesized by the direct signal and strong reflected signal from water surface. The interference signal shows periodic fluctuations in the SNR series, which is the data we need to focus on, as shown in Figure 1b. The data processing of GNSS-IR method is based on a single SNR arc of a satellite, such as the SNR data shown in Figure 1b and Figure 4 below.

In this study, the range of azimuth and elevation angles is determined by the relative relationship between water area and station. As for range of elevation angles, on the one hand, the lower limit of elevation angle range is more than 5° to avoid problems due atmospheric refraction at low elevation angle. The maximum is normally set to 20° because above this threshold there will be no interference effect, which cannot be used for water level estimation. On the other hand, the elevation angle range determination is also related to the type of receiving antenna. Different antenna gains have different upper limits, so it needs to be selected according to the observed SNR data. After satisfying these two conditions, the total number of SNR oscillation period should be considered to ensure that there are sufficient periods in this range, otherwise the accuracy of spectrum analysis will be reduced. The choice of azimuth range is based on field study or image map data. It is necessary to avoid occlusion and avoid signals reflected from other reflectors. After determining the range of azimuth and elevation angles, SNR sequence can be selected accordingly. However, before proceeding to the next detrending and spectral analysis, the screened arcs should be evaluated in various aspects, such as the height angle difference corresponding to the selected SNR sequence, the number of epochs, data miss rate and so on, which are primary quality controls and are essential.

**Figure 2:**
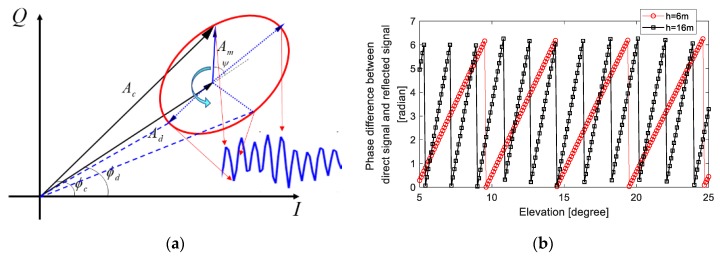
(**a**) Schematic diagram of interference signal vector decomposition; (**b**) Relationship between elevation angle and φ for two different heights.

The periodic fluctuation of SNR is explained by the vector decomposition in signal processing [24], as shown in Figure 2a. *A_c_* is the composite signal vector received by the antenna, *A_d_* and *A_m_* represent the direct signal and the reflected signal vector, respectively. The vector length indicates the strength of the signal, and the angle with the *I* axis represents the phase of the signal. After a geometric derivation [12], the phase difference between the reflected signal and the direct signal is: *ϕ* = 4*πh*sin(*θ*)/*λ*, where *h* is the vertical distance between the antenna phase center and the reflecting surface, *θ* is the satellite elevation angle, and *λ* is the wavelength of the received signal. When the reflecting surface is stationary, i.e., *h* fixed, the *ϕ* will change with the elevation angle of the satellite. It can be seen from the vector decomposition diagram that *A_m_* will exhibit a circular motion with the change of the elevation angle. When the satellite rises, *A_m_* will move counterclockwise with the starting point as the central axis. Since *A_c_* is the vector synthesis of *A_d_* and *A_m_*, the amplitude and phase of *A_c_* would change with the elevation angle. When *ϕ* changes from 0 to *π*, the change in the SNR (dSNR) will decrease, whereas when it changes from *π* to 2*π*, the *dSNR* will increase. The change rate of *ϕ* is related to *h*. The larger *h* is, the faster the change will be. As shown in Figure 2b, when *h* is 6 m, the frequency of change is significantly lower than that for *h* is 16 m. As the elevation angle changes continuously, the *dSNR* will produce continuous periodic oscillations. It is obvious that the frequency of the oscillation is positively correlated with the height *h*, so the height *h* can be determined by obtaining the frequency.

## 3. Test Sites and Data Analysis

Two test sites are selected for this study. One is Shuangwangcheng Reservoir in China, and the other is the BRST station in France. The former is for the determination of water levels in a reservoir, and the latter is for the determination of sea level changes, both are representative.

**Figure 3:**
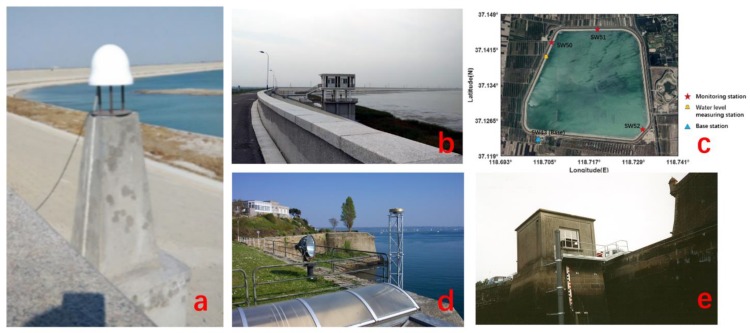
(**a**) Deformation monitoring station with radome; (**b**)Water level station at Shuangwangcheng Reservoir; (**c**) Dam monitoring stations and an overview of the reservoir; (**d**) The BRST station at the Atlantic coast of France; (**e**) Tide station near BRST station.

Shuangwangcheng Reservoir is located in Shandong Province, China [25]. The reservoir was created by a dam, which has 9.636 km long and 12.5 m high. It has a maximum storage capacity of 61.5 million cubic meters. It is an important reservoir in the Main Line Project of the East Route of the South-to-North Water Transfer Project. In order to monitor the stability of the dam, three GNSS monitoring stations and one reference station have been set up, as shown in Figure 3c, of which SW43 is the reference station, 300 m away from the dam. The other three are monitoring stations located on the top of the dam and their antennas are equipped with protective covers, as shown in Figure 3a. There is a water level measuring station near the SW50 site as shown in Figure 3b.

The water levels are measured manually at 8:00 am every day. In the dry season, the water level is about 5 m, the horizontal distance of the GNSS antenna is about 45 m from the water-land boundary. In the rainy season, the water level can reach 11.5 m, and the difference between the highest and lowest water levels can reach 7 m. We collected the observation data at three monitoring stations from May 1, to December 31, 2017 with a sampling rate of 15 s. The water levels were also collected in the same period of time. The collected water levels are used as the reference for the assessment of the GNSS-IR results. The BRST station is located on the Atlantic coast of France with the height of 13–20 m above the sea surface. The daily sea levels near the station change greatly, reaching 7 m. It is a challenging task to test if GNSS-IR technology can accurately and effectively determine sea level changes. In addition, the station has a limited scope of effective reflecting sea surface, only a sector of 60° in azimuth direction, which greatly limits the amount of the received data. We collected the observations from February 15 to February 28, 2018. Since the station can receive the data from the four satellite navigation systems, to save data storage capacity the publically assessable data are those with a sampling rate of 30 s. There is a tide gauge near the station, about 300 m away, which has long-term tidal observations with the sampling rate of one minute. The tide-gauge derived sea levels can be used as the references to evaluate the GNSS-IR results.

**Figure 4:**
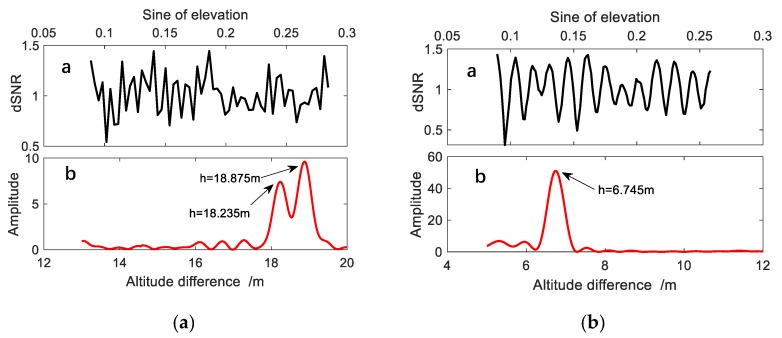
(**a**) *dSNR* and spectrum analysis results of single SNR sequence at BRST station; (**b**) *dSNR* and spectrum analysis results of reservoir station.

The data sampling by the receiver is uniform, but the change of the elevation angle of a satellite is non-uniform. Therefore, to obtain the frequency information in the *dSNR*, the Lomb-Scargle (L-S) periodogram [26] analysis method is used. The method is proved to be very effective for discrete data series and can accurately extract the frequency information in *dSNR*, as shown in Figure 4. Figure 4a shows the results of an orbital arc segment at BRST station. Due to the poor environments mentioned above, i.e., narrow scope in azimuth direction and large vertical difference between the antenna and sea level, the sampling rate of 30 s is not enough for accurately measuring sea surface. Because, the collected data sometimes cannot satisfy the Nyquist sampling theorem, the frequency obtained through spectral analysis will be inaccurate. Because of low sampling rate in this case, the *dSNR* sequence, after the tread is removed, does not show clearly any periodicity. The periodogram shows multiple peaks with similar magnitude, resulting in the difficulty in the determination of a correct frequency. While in the reservoir case the environments are much better: the height of the antenna above the water surface is 5–12 m, the sampling rate 15 s, wider scope of 120° in azimuth direction, and calm water surface. Therefore, it is easy to identify the periodicity. The periodogram is clean, less noise, and close to the ideal environment which is beneficial for the verification of GNSS-IR technology.

The results of the L-S periodogram analysis shows the maximum amplitude in the periodogram of the BRST station is much smaller than that of the Shuangwangcheng Reservoir station, and there is a lot of background noise in the BRST periodogram. In some orbital arcs, especially at low water levels, the periodogram of the BRST station is terrible. Basically, the traditional method can’t get effective results, even any correct frequency information, because of the strong noise. However, the change in sea levels due to tidal effect can be predicted which can have a great help in sea level determination with GNSS-IR technique. Although precise prediction is impossible, its integration with GNSS-IR technology, can play an important role in the determination of sea level changes in complex environments. However, the change of the water level in a reservoir is different every year. The main factors are the change of weather and artificial disturbance. When the rain is abundant, the water level rises rapidly. When the water level exceeds a warning level, flooding will occur. In the summer of dry season, due to the evaporation resulted from hot weather and the irrigation of farmlands the water level will be reduced. Therefore, it is necessary to accurately sense the change of water level by other means. We can take full advantage of the existing GNSS stations on the dam, which is originally designed for deformation monitoring purpose, to get real-time GNSS observations for measuring the water levels with GNSS-IR technology.

## 4. Quality Control Methods in Periodogram

For the measurement of water level changes for the above two conditions, the dynamic changes of the water surface and the GNSS station environment play a decisive role in the results. The response of different frequency bands to the environment is very complex, so the quality control is a critical step in a harsh environment. After initial multi-faceted quality control, the impact of the selected SNR arc on the results has been reduced to a minimum, and the quality control of the periodogram is particularly important. Screening the results according to the characteristics of the periodogram is a controllable process. The stricter the quality control is, the less effective results will be obtained, the temporal resolution will decrease, and the accuracy will be improved. There are a few more points to note before quality control of the periodogram. As introduced in the principle section, preliminary quality control is required before the periodogram quality control. In order to perform spectrum analysis efficiently, the SNR arcs obtained requires multiple evaluations, and the final *dSNR* sequence can be obtained by removing the trend item by a secondary fit. The quality control of the periodogram is based on the pre-processing of the data in the early stage.

Although the looser quality control can get more effective results, the accuracy is difficult to meet the requirements, especially for some cases, like BRST site. The classical LSP analysis methods require a minimum sampling rate. However, in the case of BRST site, the Nyquist sampling theorem may not be satisfied in spectrum analysis, so that the correct frequency cannot be obtained. Even if the sampling rate is close to the minimum of the Nyquist sampling frequency, there will be multiple similar peaks in the periodogram. If the quality control is not carried out properly, the correct frequency may be treated as noise and rejected. Therefore, it is very important to perform proper quality control, especially in the cases of poor environment of a station. There are three methods used in the analysis of periodogram to determine the correct frequency. The analysis of the periodogram is mainly based on the ratio or amplitude. The ratio here can be divided into two types. The first type of ratio refers to the ratio of the amplitude corresponding to the highest peak to the average of all other amplitudes within an allowable range of the height difference [27], as shown in Equation (1):

(1) Ratio1=AMPmax[h1,h2]MEAN(AMP[h1,h2])

where *AMP*^[*h*1,*h*2]^ is the amplitude sequence of the peaks in the periodogram obtained by the spectrum analysis between the effective altitude differences h1 and h2, and $AMP_{max}^{\left[h_{1},h_{2}\right]}$ is the maximum value in the sequence. If the value of the ratio is larger than a pre-set threshold, the frequency corresponding to the maximum value is selected as correct one. This method has been proved to be an effective quality control by many experiments. The method not only ensures accurate frequency extraction, but also effectively reduces the influence of other peaks. However, the use of the Ratio1 as quality control is sometimes problematic, particularly in a complex environment. One of the reasons is that when the quality of *dSNR* is poor, and LSP spectrum analysis may produce several peaks of similar amplitude in the periodogram. The frequencies corresponding to these similar peaks should be considered as the candidates, rather than the maximum one only. If this method is used for quality control, the correct frequency may be rejected and while a wrong one selected. Although one can increase the threshold to improve the overall accuracy, it is at the expense of many effective results. Therefore, this quality control method cannot produce a good result in the determination of the sea level change near the BRST site, many gross errors cannot be deleted automatically, and many useful results are also eliminated.

The second type of ratio, though not often used, is defined as:

(2) Ratio2=AMPmax[h1,h2]SECMAX(AMP[h1,h2])

where the *SECMAX* function is for the amplitude of the second higher peak in the periodogram. That is the ratio of the maximum amplitude to the second maximum amplitude. Unlike the first one, this method is sensitive to large noises. To some extent, Ratio2 can be considered as a kind of credibility of the result. The larger the value of Ratio2 is, the higher the credibility will be. An ideal case is there is only a single dominant peak in the periodogram, e.g., the spectral results shown in Figure 4b, Ratio2 become very large. When Ratio2 is close to 1, the reliability is the lowest. The first type of ratio is sensitive to the overall trend. If there are only two obvious peaks, the value of Ratio1 may be large, but the result is not very reliable. The reason is that when both peaks are prominent, the correct one cannot be judged only by the magnitude of the amplitude.

For the quality control we can also use the amplitude only. The magnitude of an amplitude is related to the significance of the corresponding frequency. The larger the relative amplitude is, the stronger the significance of the corresponding frequency will be. The third type of ratio is defined as:

(3) $$Ratio3=AMP_{max}^{\left[h1,h2\right]}$$

## 5. Quality Control Results

In the data processing, after setting the thresholds of the three parameters, if the values of Ratio1, Ratio2, and Ratio3 of the periodogram does not exceed the set threshold, the results will be discarded.

**Figure 5:**
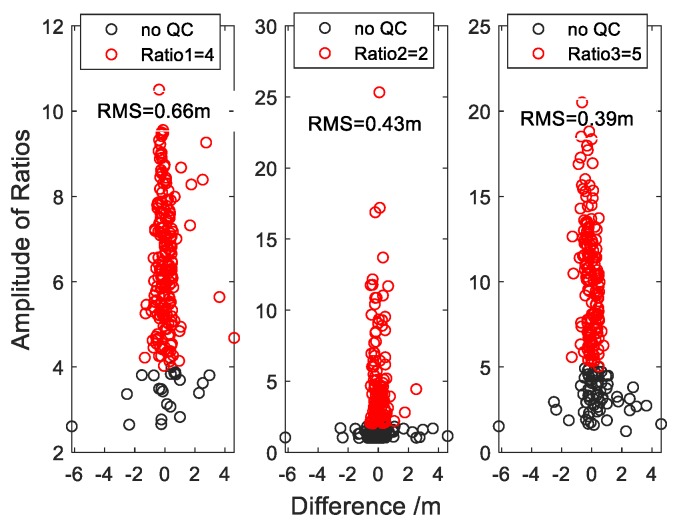
The relationship between three ratios and the result errors. The X axis represents the difference between the measurements of the tidal station and the water level results obtained by GNSS-IR technique.

Figure 5 shows the relationship between the selected thresholds of the above three methods of quality control and the differences between the derived results and the actual measurements for the case of BRST station. The black scattered points (partially covered by the red ones) are the results without any quality control. All scattered points are basically distributed near the vertical line of 0 m. Some scattered points deviate farther, and the RMS is 1.132 m, indicating poor accuracy. From Figure 5, we can find that the general trend of the three scatter diagrams is roughly consistent, that is, the scatter with large error is at the bottom, and the scatter with small error is at the top, which means that we can achieve the quality control effect by setting the threshold of these quantization parameters. In addition, there are some differences in detail between the upper and lower parts. The results of the two methods of quality control, Ratio1 and Ratio3, are similar. The lower part of the scattered points has some unexpected gross errors, and the upper part still has a few, especially for the method of Ratio1. Therefore, setting of a certain threshold can only improve the overall results but cannot effectively eliminate gross errors in the upper part. For example, as shown in the red scattered points in Figure 5, when the threshold of Ratio1 is 4, some gross errors exist, and the RMS is 0.66 m. When the threshold of Ratio3 is 5, the gross errors are almost eliminated, but the overall accuracy with RMS being 0.39 m, needs to be improved. The distribution of gloss errors for the method of Ratio2 is slightly different. The upper part has relatively few gross errors, and the scattered points are mainly concentrated near the vertical line of 0 m. In the bottom there are many large errors, but also a lot of good results. In contrast, the Ratio2 method controls the periodogram more strictly. When the threshold of Ratio2 is larger, the result is more reliable and accurate, but the number of the valid results is smaller. When the threshold of Ratio2 is 2, the number of valid results is 52% of the total number of the data source (SNR series), and the RMS is 0.43 m. Although a lot of gross errors are eliminated, a large number of valid results at the bottom are also eliminated, which is a drawback of this method. For the coastal sites, like the BRST station, with complex environments, the next section will discuss a possible solution by an integration with tidal prediction. In addition, due to the various factors at different stations the methods of Ratio1 and Ratio3 have different order of magnitude, therefore some difficulties will occur in determining their threshold.

**Figure 6:**
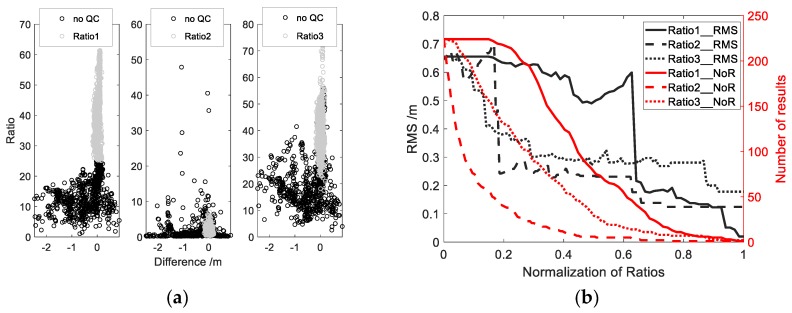
(**a**) Errors at reservoir dam monitoring station and the relationship among three ratios. The X axis represents the difference between the measurements of the water level station and the results obtained by GNSS-IR technique; (**b**) Comparison of RMS and number of results for three quality control methods, NoR is a shorthand for number of results.

The three ratios are defined differently, and their impacts on the results will be different. Figure 6b shows the results using three methods of quality control for the BRST station. Because of the different magnitude of the three quantization parameters. In order to better compare the differences between the three quantization parameters used in quality control, normalization is carried out according to the range of quantization parameters. The horizontal axis is the normalized thresholds, from 0 to 1. The three black lines represent the RMS of the derived water level with different thresholds, and the three red lines represent the relevant number of the final valid results. As the threshold of Ratio1 increases, the RMS decreases slowly in the beginning, and sometime even increasing a bit. The reason for the increase is that many gross errors are not eliminated, and some good results are deleted. When the normalized threshold approaches 0.65, the RMS drops sharply, which is due to the elimination of many gross errors, but the number of the results decreases slowly. This is an advantage of the method, over the other two. The RMS with the methods of the Ratio2 and Ratio3 are generally consistent. Due to the elimination of many gross errors in the beginning, the RMS decline faster, and the number of the results is reduced quickly as well, especially for the method of Ratio2, which suggests these two methods of the quality control are at extreme. In the process of improving the accuracy, many gross errors are eliminated, and a lot of better results are deleted as well.

In the case of a better environment, like the reservoir, the results are shown in Figure 6. It is obvious from Figure 6a that threshold setting for the Ratio1 can better separate the gross errors and the accurate results. When the threshold of Ratio3 is set to about 45, the similar results as the method of Ratio1 can be achieved, but many accurate results are deleted as well. Because there is no absolute relationship between the magnitude of the amplitude and the accuracy in the case of a single peak, it is not proper to use the amplitude as a criterion for judging if the result is accurate. But it can be treated as an additional parameter to assist the judgment. The gray scattered points in Figure 6a are the results when the threshold of Ratio1 is set to 7. The scattered points are around the vertical line of 0 m, almost all the gross errors are eliminated, and a better accuracy with RMS 5.6 cm is achieved. The threshold setting for the method of Ratio2 is the worst, which cannot eliminate all the gross errors, because there is only one obvious peak in the periodogram. and the frequency corresponding to the peak can be considered no gross errors and highly reliable. The main concern in this case is not the extraction of the correct frequency, but other factors, which affect the overall quality of the periodogram, leading a lower accuracy. These factors include the fluctuations of the water surface, the change of the reflecting surface, and other unknowns. Therefore, the threshold setting for the method of Ratio1 is superior to Ratio2. This is because the threshold setting for the method of Ratio1 is sensitive to the overall quality of the periodogram, while for Ratio2, it is mainly for the local part of the periodogram. The method of Ratio2 is more for reliability measure. It is suitable for evaluating the reliability of the results under the situation that the outcomes of spectrum analysis are not optimistic. When the reliability of the results is high, the value of Ratio2 will not be important, and the distribution of scattered points will not like Figure 5, but Figure 6a, which shows an irregular black cluster of scattered points. And the bias corresponding to a large threshold for Ratio2 will not be the smallest, and threshold setting cannot achieve a good result. Moreover, in the left part of the second plot in Figure 6a, there is a second gathering of black scattered points. This is mainly caused by a platform. In the design of the reservoir dam, a platform of 15 m wide is built in the middle of the dam to alleviate the slope of the dam. When the platform is above the water level, the partial reflected signal is not from the water surface but the platform. The result is the altitude difference between the antenna and the platform.

## 6. Guidance-Based Quality Control Strategy 

### 6.1. Quality Control Based on Tidal Prediction Guidance (TPG)

As mentioned above, the method of Ratio2 is suitable for the cases of poor environments. A large threshold can easily remove the large gross errors, but sometimes also eliminate the good results with small errors due to the double peaks in the periodogram. This phenomenon is ubiquitous. In addition, the threshold setting for the method of Ratio1 cannot completely eliminate the gross error, but improve the overall accuracy.

An integration of the above three methods can take full advantage of the merits of each method and should be a good choice for improving the efficiency of quality control. Another method of quality control is based on tidal prediction guidance (TPG), which will be introduced below.

Based on the long-term measurements of a tide gauge, the tidal coefficients can be estimated. The sea level at any time can be predicted with these coefficients. The paper [14] used the tidal coefficients to correct the errors caused by the dynamic change of the reflecting surface. which has been proved to be effective. In a least-squares tidal analysis, tidal coefficients can be obtained by Equation (4):

(4) $$\bar{h}=\sum_{i=1}^{N}C_{i}f_{i}\cos\left(\omega_{i}t+\upsilon_{i}\right)+S_{i}f_{i}\sin\left(\omega_{i}t+\upsilon_{i}\right)$$

where *S_i_*, *C_i_* are sine and cosine coefficients of *N* independent tidal frequencies *ω_i_*, *N* is the length of results series, *f_i_*, *υ_i_* are the nodal amplitude factors and equilibrium phases, $\bar{h}$ is the result of GNSS-IR. After a long-term tidal series is obtained by GNSS -IR technique with historical SNR observations, recent approximate water levels can be predicted. For the calculation of tidal wave coefficients, the length of historical data plays a key role in the accuracy of the coefficient calculation. The more historical data, the better the prediction effect. In addition, GNSS stations usually have been established for a long time, far more than one year. There are enough historical data to invert the tidal wave coefficients. If the data quality is good enough and combined with the characteristics of tidal changes, one month’s tidal change data can well simulate the characteristics of tidal changes. In this paper, one-year historical water level data obtained by GNSS-IR technique are used to retrieve sea level changes near BRST stations to estimate the tidal wave coefficients.

**Figure 7:**
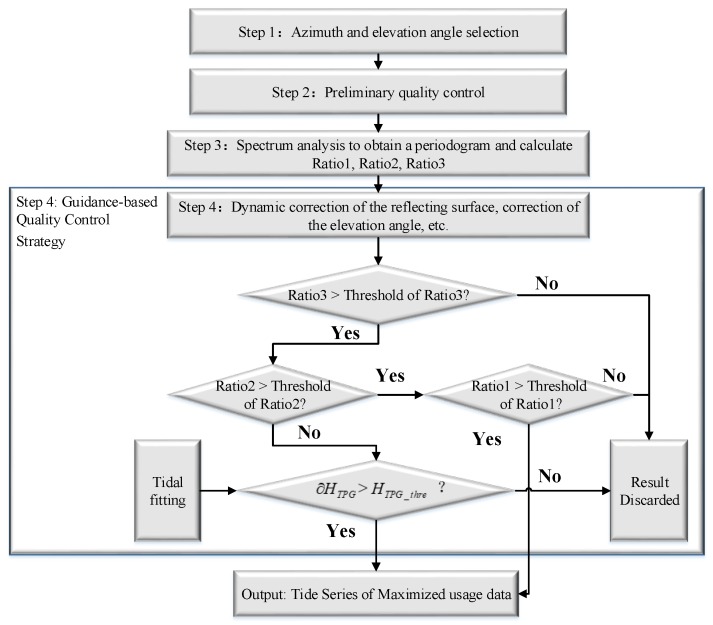
Flow chart of the TPG method.

The TPG method is to solve the uncertainty of the multi-peaks in a periodogram under complex conditions. The three quantitative parameters for periodogram play an auxiliary role in the periodogram quality control. The periodograms can be effectively classified by the combination of the three parameters: (1) There is only one dominant peak, which is easy to extract the correct frequency. (2) With multiple dominant peaks and approximate spectrum power, it is necessary to use external control to guide frequency selection. (3) The third type periodogram is completely unnecessary for further analysis, which is characterized by low overall power, and no dominant peak, need to be abandoned. Both the first and the third type periodograms are easy to deal with, but for the second, further judgment and treatment are needed. The main idea is to select the correct frequency among the multiple frequencies with similar peak amplitudes with assistance of a predicted value, rather than direct use the one with the maximum amplitude. When the predicted value is close to the frequency corresponding to one of the peaks, it can be considered that one may be of interest. As shown in Figure 9a, there are multiple peaks in the periodogram, such as peak1, peak2, peak3, all of which are contaminated by noises due to the poor quality of the data source. Combined with the measured data, it is found that the accurate result is not the frequency corresponding to peak1, but the frequency corresponding to peak3. In this special case, we can introduce an external control to extract the correct frequency. We can compare the derived sea levels corresponding to the peaks with the predicted sea level, and select the one as the correct result, which has the smallest bias with the predicted value. Of course, an allowable bias also needs to be controlled within a certain range, as shown in Figure 7.

(5) $$\partial H_{TPG}=\min(\left|H_{predicted}-{\bar{h}}_{peak1}\right|,\left|H_{predicted}-{\bar{h}}_{peak2}\right|,\left|H_{predicted}-{\bar{h}}_{peak3}\right|)$$

where *H_predicted_* is the predicted water level according to Equation (4), ${\bar{h}}_{peaki},i=1,2,3,\ldots ,$ is the estimated results according to multiple frequencies with similar peak amplitudes in periodogram.$\partial H_{TPG}$ is the bias between prediction and estimated results. And, we need to set *H_TPG_thre_*, a threshold of $\partial H_{TPG}$, to control the range of errors. The flow chart of TPG is shown in Figure 7.

As shown in Figure 7, the three evaluation parameters of periodogram work synergistically. The Ratio1 and Ratio3 can control the overall quality of the periodogram. When the values of Ratio1 and Ratio2 are lower than a limit, there is no need for further analysis. When the value of Ratio2 is lower than the threshold, the predicted sea level is used as a guide for correct frequency extraction, which can improve the utilization of the data and increase the temporal resolution.

**Figure 8:**
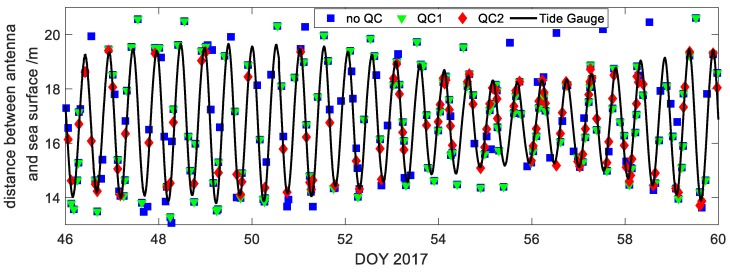
Comparison of different quality control methods for water level inversion at BRST Station. No QC means no quality control, QC1 refers to the threshold setting method of Ratio1, QC2 refers to the TPG method. The solid black line represents the records of tide gauge.

**Figure 9:**
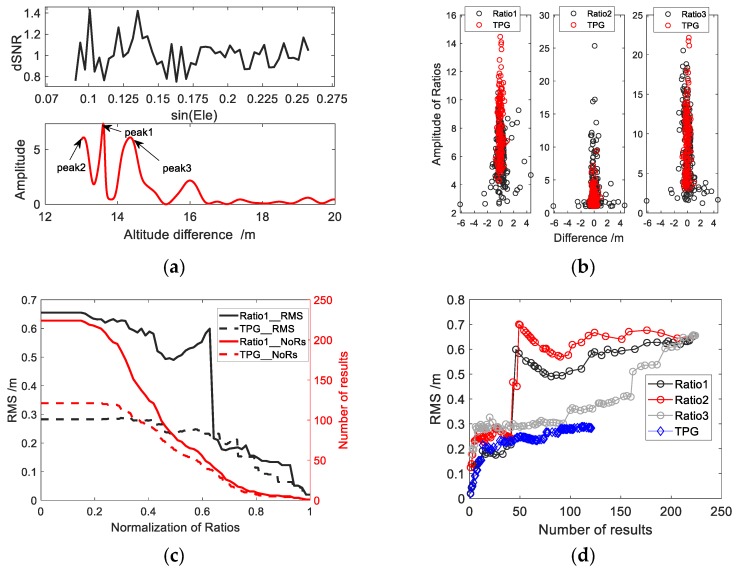
(**a**) In the case of multiple peaks, the frequency corresponding to the maximum amplitude is not the correct value; (**b**) Results after adding TPG quality control method at BRST station. The X axis represents the difference between the measurements of the tidal station and the water level results obtained by GNSS-IR technique; (**c**) Comparison of TPG and Ratio1 threshold settings for two quality control methods; (**d**) Comparison of TPG and three threshold setting quality control methods with respect to accuracy and number of results.

We analyzed the data at BRST station as an example. Figure 8 shows the results of water level inversion obtained by three data processing methods. QC1 method cannot remove all outliers that are caused by periodogram with multiple dominant peaks as shown in Figure 9a. A simple threshold setting method cannot effectively obtain accurate results, but will get outliers. TPG method eliminates all outliers and corrects them to correct results. The red scattered points in Figure 9b are the results of adding the TPG method in the quality control, and the black scattered points are the results without TPG in quality control. It is obvious that the red scattered points are distributed inside the area of the black scattered points, indicating the elimination of a lot of gross errors, and there is no node stratification as shown in Figure 6a left, which deleted the accurate results in the lower part, and reduced data utilization. It can be seen from Figure 9c that the method of adding TPG removes the gross errors directly, and when the threshold of the TPG is set to the minimum, the initial RMS is within 30 cm. If only the method of Ratio1 is used for quality control, to achieve the same accuracy the threshold needs to be set to 0.65 (after normalization), and the number of the results is less than 50. While for the TPG method the number of results is about 120, increasing by nearly 140%.

Figure 9d is a comparison among the four methods of the quality control. The blue diamond curve shows the number of results and RMS for the TPG method. The black, red, and gray scattered points are for the methods of Ratio1, Ratio2, and Ratio3, respectively. For the same number of valid results, the accuracy of the traditional three methods is different. When the number of the valid results is less than 50, the method of Ratio1 works best, and Ratio2 and Ratio3 are similar, but when the number of the results is greater than 50, the RMS for the methods of Ratio1 and Ratio2 suddenly increases. On the contrary, RMS for the method of Ratio3 is still around 30 cm with the number of the results between 50 and 100, and starts to increase slowly after 100. Obviously, if the accuracy requirement is not high, the threshold setting method of Ratio3 is optimal. Compared with the three methods of Ratio1, Ratio2, and Ratio3, the TPG method demonstrates its merit of accuracy for the same number of results. When the number of the valid results is less than 50, its performance is similar to that of the Ratio1 method. When the number of the results is greater than 50, the performance of the TPG method is better than that of Ratio3, and the accuracy is always the highest and in a similar order of magnitude. For example, when the number of the results is 100, the accuracy with the TPG method is almost 100% higher than that with the method of Ratio1. This demonstrates the effectiveness and efficiency of the TPG quality control method, especially in the harsh conditions around a GNSS station.

### 6.2. Quality Control Based on A Single High-Quality Satellite

For good conditions, GNSS-IR can achieve high accuracy, but various uncontrollable factors will generate a lot of noise, and there also exist difficulties in setting the thresholds for the methods of quality control. Unlike sea level, the water level of inland waters cannot be predicted through long-term observations, but good environment at a GNSS station is of advantage. The RMS of the GNSS-IR derived results can be smaller than 5 cm, at least a half of the RMS of the determined sea level. In addition, the water level change of a reservoir is completely different from the change of sea level, which has great and daily periodical variations. For example, the sea level near the BRST station change by 7 m twice a day. On the contrary, the daily variation of the water level of a lake or reservoir is quite small. Under the normal circumstances, the daily variation of the Shuangwangcheng Reservoir is smaller than 10 cm. The analysis of the GNSS data of a station on the dam of the Shuangwangcheng Reservoir tells the accuracy from a single satellite can reach 5 cm. Since the orbiting period of a GPS satellite is 11 hours and 58 minutes, the observation of a single satellite can give one effective result every day for a long period of time in a suitable scope of azimuth. Because of little change in the environment, the daily results can be used to get the overall trend of the water level changes, which can be introduced as an external constraint for quality control. Similar to the TPG method, all the results before a new measurement are used to fit the trend of the water level changes, which is used as the external control. The difficulty lies in the choice of a single high-quality satellite. Generally, it is necessary to consider the sky-plot of satellite and the field environment of the station to select the high-quality arc, which will contain less noise, and there will be an obvious and dominant peak in the periodogram. Single satellite is chosen because the orbit of GPS satellite is very close every day, so the environment of monitoring water level and the position of specular point will not change greatly. Under these excellent conditions, the daily results are stable, unlike multiple satellites inversion strategy, because one or more satellite signals are affected by various poor environments and many outliers appear, which is not conducive to the expression of water level trend and the establishment of external control conditions. One thing to note about using GQCS is that it does not necessarily require high accuracy as an external control, but high continuity and stability are required. Therefore, we should choose a single high-quality satellite that can obtain stable results as a data source for external control, instead of using strategy of multi-satellite fitting.

**Figure 10:**
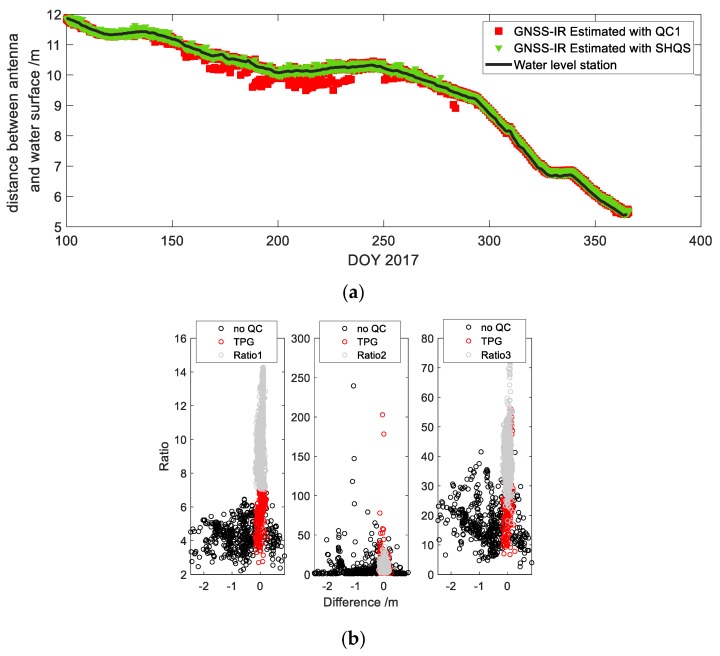
(**a**) Comparison of different quality control methods for water level inversion at Shuangwangcheng Reservoir. QC1 refers to the threshold setting method of Ratio1. The solid black line represents the records of water level station. (**b**)The results of the water level at a station on the reservoir dam with different methods of quality control.

Figure 10 shows the results for the reservoir case. In Figure 10a, the red scatter represents the inversion results obtained by the method of QC1, while the green scatter shows the results obtained by the method of GQCS. It is obviously found that, it is obviously found that there are many outliers at the time of DOY 200. However, the water level obtained by GQCS method is close to the record of water level station, and the overall accuracy can reach 5 cm.

In Figure 10b, the black scattered points are the errors without quality control, the gray scattered points are the result with the threshold of Ratio1 being 7, and the red scattered points are the result with the quality control based on single high-quality satellite. GPS PRN03 satellite was selected as the high-quality satellite. The RMS of the results is 6.1 cm, which is enough to get the trend of the overall water level changes. For a comparison, when the threshold of Ratio1 is 7, the number of the results is 3004 out of the total number 3810, eliminating almost all the gross errors with RMS of 5.9 cm. However, due to the node stratification, the good results below the threshold have not been considered. At the same time, it is found that the good results for the methods of Ratio2 and Ratio3 are also deleted. The quality control based on a single high-quality satellite compensates for this shortcoming. As shown by the distribution of the red scattered points, the number of the valid results obtained is 3379 with RMS 6.1 cm. With similar accuracy this method takes not only the results selected by the method of Ratio1, but also the results below the threshold of 7, which improves the data utilization rate and the temporal resolution.

## 7. Conclusions

The GNSS-IR is a new technique to determine water levels and the results are significantly affected by the environment around a GNSS station. There are two typical cases: one is inland waters, like lakes and reservoirs, and the other is sea. They have different characteristics. For the case of sea level determination, the accuracy due to the great change of the sea surface and the poor environment around a GNSS station is difficult to meet the requirements. Like the BRST station, not only the scope of the azimuth direction is small, but also the vertical distance between the reflecting sea surface and the GNSS antenna is beyond a normal level. It is difficult to obtain high-precision results when the sampling rate is insufficient. For inland waters, due to the favorable station environment and the relative calm of the water surface, the RMS of the single result estimated by the GNSS-IR can be within 5 cm. This paper takes the BRST station in France and the deformation monitoring station on the Shuangwangcheng Reservoir dam as the representative examples to discuss the different considerations on water level determination.

In addition, we focus on analyzing the influence of different methods of quality control in periodogram, and the differences among the three methods of quality control are explained. There is not fully adapt parameter to deal with all kinds of environments. Although Ratio1 parameter is widely used and can work well in normal circumstances, it has drawbacks in complex environments. The Ratio3 is also an important parameter. The overall amplitude of the periodogram is directly affected by the quality of the environment. Small amplitude indicates poor quality data, and the period in SNR will be not significant, which is an important auxiliary parameter. For Ratio2, which is not commonly used, this parameter can be considered as the reliability of a result. The smaller the value, the lower the unreliability of the results will be. When there are two peaks of the same amplitude, it is difficult to confirm which frequency is correct. Based on this parameter, this paper proposes the Guidance-based Quality Control Strategy (GQCS). When Ratio2 is close to 1, it means that there are at least two peaks in the periodogram with approximate amplitudes. At this time, we cannot simply assume that the frequency corresponding to the maximum peak is correct. It is possible that the frequency corresponding to the secondary peak is correct due to undetermined factors, and it is found that this situation is ubiquitous, especially at BRST station. In this special periodogram, the Ratio1 method is invalid, because of the precondition that the frequency corresponding to the maximum peak is correct, so it is necessary to introduction the Ratio2 parameter. In order to determine the correct results among the plurality of approximate peaks, external constraint plays a guiding role. The Guidance-based Quality Control Strategy proposed can be applied to two representative environments. At the coastal station, the historical observation data can be used to fit the water level to predict the recent changes and serves as a guide for sea level monitoring. For high-quality environments such as reservoirs, the stability characteristics of single satellite measurements can be utilized to guide the flexible processing of complex periodogram. The experiments demonstrate that the Guidance-based Quality Control Strategy can improve the accuracy of the results and data utilization.
